# Structural and chemical heterogeneity of pulp stones in young untreated bovine incisors: frequent dentin-like mineralizations

**DOI:** 10.1016/j.yjsbx.2026.100151

**Published:** 2026-07-06

**Authors:** Hamza Elfarraj, Oleksandra Marushchenko, Franco Lizzi, Leona J. Bauer, Ivo Zizak, Hagay Shemesh, Ioanna Mantouvalou, Paul Zaslansky

**Affiliations:** a Department for Operative, Preventive and Pediatric Dentistry, Charité – Universitätsmedizin Berlin, Aßmannshauser Straße 4-6, 14197 Berlin, Germany; b Helmholtz-Zentrum Berlin für Materialien und Energie, SyncLab (PS-GSYN), Albert-Einstein-Straße 15, 12489 Berlin, Germany; c Institute of Physics and Astronomy, Technical University of Berlin, Hardenbergstr. 36, 10623 Berlin, Germany; d Berlin Laboratory for Innovative X-ray Technologies – BLiX, Germany; e Helmholtz-Zentrum Berlin, Department for Structure and Dynamics of Energy Materials (SE-ASD), Albert-Einstein-Straße 15, 12489 Berlin, Germany; f Department of Endodontology, Academic Centre for Dentistry Amsterdam (ACTA), Amsterdam, Netherlands

**Keywords:** Bovine teeth, Intact healthy non-aged dentin, Laminography, XRD, XRF, SEM-EDX, Ectopic mineralization, Apatite

## Abstract

Pulp stone research has predominantly relied on aged or clinically treated human teeth, limiting insights into early-stage mineralization processes. This study investigates young bovine incisors (3–4 years old, ≤2 years after eruption) as models for characterizing the prevalence, morphological, and material characteristics of ectopic mineralized deposits frequently found within the dentin–pulp complex. A total of 100 incisors, selected from a pool of 1000 teeth extracted from 250 lower cattle jaws (*Bos* Taurus Taurus), were screened using cone-beam computed tomography (CBCT). Teeth exhibiting radiopaque deposits within the pulp space were further analyzed by high-resolution micro-computed tomography (μCT). A subset of specimens underwent complementary multimodal characterization, including laminography, scanning electron microscopy with energy-dispersive X-ray spectroscopy (SEM–EDX), micro-X-ray fluorescence (μ-XRF), and X-ray diffraction (XRD). Adjacent healthy dentin served as a reference. Mineralized deposits were identified in the pulp spaces of 30 % of the seemingly normal teeth, appearing as free-standing structures or, more frequently, larger deposits attached to canal walls. μCT demonstrated substantial variability in size and spatial distribution, with volumes ranging from <0.01 mm^3^ to >5 mm^3^. SEM and X-ray imaging revealed heterogeneous internal architectures, including layered and channel-like features. μ-XRF analysis identified localized zinc enrichment, while XRD indicated hydroxyapatite as the dominant crystalline phase, with lattice parameters comparable to adjacent dentin. These findings highlight the morphological heterogeneity of mineralizations in young bovine teeth, though the present study does not directly reveal causative mechanisms. Given the absence of age-related and pathological confounders, bovine incisors represent a useful experimental model for investigating early-stage, partially controlled biomineralization of the extracellular matrix in the pulp of healthy normal teeth.

## Highlights

• Approximately 30% of permanent caries-free incisors of Bavarian diary/meat cows contain pulp stones.
• Many stones are attached to dentin walls and they contain patches of dentinal tubules suggesting development during root formation.
• Coronal stones are larger and seemingly structurally homogenous.
• Radicular stones are smaller and often contain large heterogeneous mineral/organic layers/inclusions.
• Pulp stones in young bovine teeth exhibit significant zinc enrichment at their peripheries.

## Graphical abstract

**Figure:**
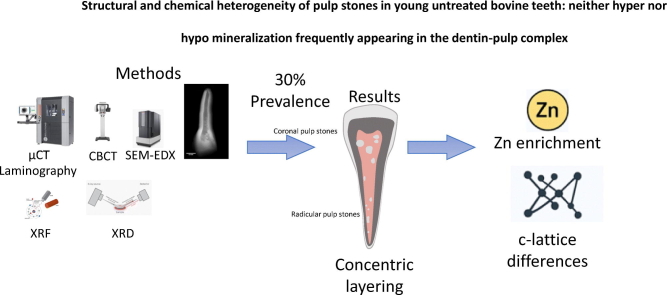

## 1 Introduction

Mammalian teeth share many commonalities and evolved to become impressively resilient and intricately structured mastication tools: each tooth is supported by the periodontal complex and has well described functional properties (Nanci, 2007). Structurally, all teeth are covered externally by enamel, in the crown, a material that is highly mineralized and relatively thin. The crown is supported by dentin, a resilient porous trunk forming the bulk of the tooth and roots. Minor though important additional structures include thin cementum that covers the roots and the pulp soft tissue within a central cavity serving as a reservoir of nerves and vasculature (Berkovitz et al., 2024). Cementum is considered a part of the supportive periodontium, which includes also jaw-bone and the gingiva that forms a protective mucosal seal (Newman et al., 2011). The periodontium anchors teeth firmly in place via the periodontal ligament with fibers that maintain position and also transmit masticatory forces further to be dissipated by the alveolar bone and the jaw (Lai, 2017; Moga et al., 2024; Thant et al., 2022). Teeth are thus complexes of tissues, some unmineralized, some moderately mineralized and some highly mineralized that define “normal anatomy” (Nanci, 2007). Once fully formed however, and with increasing age, the living dentin pulp complex slowly closes-in over the years, through gradual, incremental, secondary-dentin deposition. This later-produced material grows inwards at the expense of the pulp space, by deposition of partially irregular, deformed dentin. However, sometimes rapid dentin-pulp complex mineralization takes place, as has been reported, for example following chemical or bacterial exposure, where reactive bio/mineralization contributes to the efforts to block influx of pathogens. Consequently reparative mineralized tissue (Goldberg and Smith, 2004; Magloire et al., 2001; Nanci, 2007), known as tertiary dentin, is often found near regions of tooth fracture or caries. In addition to all of the above, a distinct type of poorly-understood mineralization is observed only in some teeth, occasionally, appearing as localized mineralized masses known as pulp stones. Such deposits may significantly impinge on the soft tissue, occupying and sometimes even blocking the pulp space chambers completely. It is not clear if pulp stones are related to diffuse mineralization zones (He et al., 2022) as reported in some pulp tissues of a range of human teeth. Clinically, often pulp stones can be ignored, unless they obstruct and complicate treatment (e.g. root canal procedures). The appearance of such stones has been attributed to processes of aging but they might be partially-controlled possibly via mechanisms comparable to other pathological calcification spanning blockage of blood vessels (Vidavsky et al., 2021), kidney stone depositions or even cartilage mineralization, as reported e.g. in shark skeletons (Seidel et al., 2016). In particular, pulp-stone formation, timing, and cause of appearance are not known (Ten Cate, 1994). Current theories (e.g. caries or dental treatment) (Zhan et al., 2023) link pulp stones to a protective response (Berès et al., 2016), associated with chronic or long-term exposure to mild pathogenic stimulation (Goga et al., 2008). Some scholars identify relationships between pulp mineralizations and dentin/cementum permeability (He et al., 2022). Most reports however are based on observations in mature human teeth that have been in use in the oral cavity, thus exposed to many influencing factors for many years. Specifically, such teeth often include deep or slow-developing caries or may have been exposed to recurring mechanical overload (Arys et al., 1993; Demant et al., 2022; Luukko et al., 2011). To the best of our knowledge, there are no reports of animal models that can help study this enigmatic biologically-induced phenomenon.

Extracellular matrix mineralizations in the human dentin-pulp complex have been documented for decades (Chandler et al., 2003; Marshall et al., 2023) and they have been categorized as (i) diffuse mineralizations (Johnson and Bevelander, 1956), (ii) dystrophic calcifications (Goga et al., 2008) and (iii) discrete mineralization (Huang and Chen, 2016; Sayegh and Reed, 1968; Shafer et al., 2006). Pulp stones are the main example of (iii) (Goga et al., 2008), and are sometimes termed nodules (Stafne and Szabo, 1933) or denticles (Marshall et al., 2023; Moss-Salentijn and Hendricks-Klyvert, 1988). When sufficiently large, pulp stones become detectable by X-rays, observed as dense masses within the otherwise translucent pulp (He et al., 2022; Tosco et al., 2025). They can be found in coronal or root areas of the pulp, varying in shape and size, with reported diameters spanning 50 μm to several millimetres (Johnson and Bevelander, 1956; Sayegh and Reed, 1968). They may be embedded in the dentin walls, they may bulge out of dentin and remove out of dentin into the pulp-space, or they can be suspended, free-standing, in the stromal tissue (Goga et al., 2008; Johnson and Bevelander, 1956). Substantial literature describes denticles as dentin-like matrix structures found within the dentin-pulp complex. In human teeth, such denticles have classified into true denticles and false denticles. True denticles are said to be formed by terminally differentiated odontoblasts which may explain why they are bound to the dentin and appear to contain dentin tubules (Goga et al., 2008). False denticles is a term that has been given to pulp-stone structures found suspended within the pulp tissue, but in some cases, they may become bound to the dentin walls following continuous growth. It has been demonstrated that false denticles in healthy human molars are formed from odontoblast-like cells that differentiate from pulp stem cells in response to non-physiological stimuli (Korkmaz et al., 2022). We refer to all of the above as pulp stones, and they typically have oval or round (spherulitic) shapes with irregular surface morphologies with variable, poorly defined internal structural organization, as seen e.g. by scanning electron microscopy (SEM) (Le May and Kaqueler, 1991). In the case of human deciduous molars, a radial arrangement of crystals has actually been reported as observed by microradiography and polarized light microscopy (Arys et al., 1989b; Arys et al., 1989a). Yet often stones appear to resemble dentin and contain collagen fibers and dentinal tubules (Milcent et al., 2019). In that regard, chemical analysis identified the presence mainly of phosphorus (P) and calcium (Ca) by EDX with some magnesium (Mg), carbon (C), nitrogen (N), sulfur (S), while X-ray fluorescence (XRF) analysis also found notable amounts of copper (Cu), zinc (Zn), and strontium (Sr), with substantially higher Zn levels in pulp stones compared to healthy dentin (Berès et al., 2016). The mineral phases identified in stones include carbonated apatite (Aoba et al., 1980; Milcent et al., 2019; Trowbridge et al., 1996), hydroxyapatite (De Rysky, 1981; Seltzer, 1984) and brushite (Kodaka et al., 1998).

Pulp stones are commonly described as mineralized responses associated with chronic irritation in the dentin–pulp complex (Goga et al., 2008; Rajendran, 2009). However, it has been reported that they can form also in healthy human wisdom teeth (Korkmaz et al., 2022) suggesting that chronic irritation is not the only cause. Studies based on human teeth are often limited by the availability of chronologically-young specimens, because human teeth are typically obtained after prolonged functional use and pulp stone formation may reflect cumulative exposure to mechanical, microbial and environmental/clinical-treatment influences. These factors introduce multiple confounding variables, making it difficult to distinguish between early-stage physiological mineralization processes and those associated with long-term pathology, appearing via controlled or uncontrolled biomineralization processes.

In this context, the investigation of young, untreated teeth without caries or prior dental intervention is of particular interest, as such specimens may provide insight into earlier stages of pulp-associated mineralization. Bovine incisors represent a practical model system, as they are typically available within a relatively short period after eruption (≤2 years) and are generally free of restorative or pathological alterations. It has long been established that bovine incisors have chemical composition and structure that is comparable to that of human teeth (Lizzi et al., 2025), supporting their use in structural and compositional analyses (Al Ankily et al., 2026). Although reports of pulp stones in bovine teeth remain limited (Kodaka et al., 1998), this model offers several advantages, including controlled age range, consistent anatomical features, high availability from the meat industry and reduced variability related to minimal clinical treatment. These characteristics may facilitate more systematic investigation of mineralization patterns potentially contributing to a better understanding of pulp stone formation.

Bovine dentition is highly specialized for processing fibrous forage, with 6 incisors, 1 canine, 3 premolars and 3 molars in the lower jaw and 3 premolar and 3 molar in the upper jaw (Singh et al., 2017). The upper jaw lacks incisors and canines, featuring instead a keratinized dental pad that functions as the contact surface for the lower incisors during food intake (Singh et al., 2017). Mastication occurs in two phases—initial ingestion and rumination—where regurgitated food is repeatedly chewed with the molar teeth by lateral grinding movements used by the cow to reduce food-particle size (Dijkstra et al., 2005; Virot et al., 2017). These repetitive, forceful motions, combined with frequent contact between the lower incisors and the dental pad, generate substantial mechanical stress on the dentition (Johansson, 2011). Such conditions may contribute to accelerated wear potentially affecting the integrity of dental tissues, as has been reported in sheep teeth (Martins et al., 2023).

Here we study characteristics of the chance finding of substantial numbers of pulp stones in a large pool of extracted bovine incisors. These teeth experienced natural functional loading during the short animal lifetime prior to extraction and provide preliminary insights into the form, chemistry and structure. We combine cone beam computed tomography (CBCT), micro-computed tomography (μCT), laminography, scanning electron microscopy with energy-dispersive X-ray spectroscopy (SEM-EDX), micro X-ray fluorescence (μ-XRF) spectroscopy and X-ray diffraction (XRD) to study the prevalence, morphology and structure/composition of pulp stones in young bovine teeth.

## 2 Materials and methods

### 2.1 Teeth collection and storage

The 100 teeth used for this ex vivo study were randomly selected from a large collection of incisors (1000 teeth) extracted from the jaws of 250 slaughtered cows (*Bos Taurus Taurus*), approximately 4 incisors per jaw. The jaws were obtained from a commercial Bavarian supplier (FHG Henke GmbH, Feuchtwangen, Germany), originating from healthy animals aged 3–4 years and otherwise used for human consumption. All the teeth exhibited clear signs of attrition/wear on the lingual surfaces having served for feeding for up to three years of mastication. The teeth met the following inclusion criteria: morphological similarity, straight, fully developed roots with no signs of root fracture or resorption. The integrity of each single-rooted tooth was ensured before storage, fully hydrated, in 0.5% chloramine-T solution at 4 °C immediately after extraction.

The root surfaces were cleaned to remove all remaining periodontal tissues. Several of the teeth identified by μCT as containing stones in the crown or root (*n* = 8) by μCT were embedded in acrylic resin (Technovit 4071, Heraeus Kulzer, Hanau, Germany) for comparative microscopy by physical slices. To do so, cross-sectional and longitudinal sections containing the pulp stones were cut using a water-cooled diamond blade (Exakt, Advanced Technologies GmbH, Norderstedt, Germany). Each slice was subsequently polished with silicon carbide abrasive paper up to P4000 (MICROCUT, RA11, USA). Multiple cross-sections were extracted from each tooth resulting in a total of 22 physical slices, each containing some part of a pulp stone. All these slices underwent a 30-s cleaning in an ultrasonic bath and were dehydrated through a series of ethanol concentrations (25%, 50%, 75%, 96%, and 99.6%), with solution exchanges made every 24 h. Thereafter, the slices were dried in a silica gel desiccator for 48 h. Two of these slices designated for X-ray transmission measurements, were further polished on both sides and air dried, to create thin parallel slivers approximately 200 μm thick. For all comparative analyses, adjacent dentin from each section served as control, to ensure comparison to normal with identical mineralization history, age, and environmental exposure. No animals were euthanized explicitly for this study. For imaging-based prevalence and morphometric characterization, each individual tooth served as the observational unit, as each tooth was independently screened and segmented. Because more than one tooth may have originated from the same animal (chances of 1:10 given the 100 teeth taken from the pool of 1000), potential within-animal clustering cannot be excluded.

### 2.2 Cone beam computed tomography - CBCT

All 100 teeth were scanned using a Morita Veraviewepocs 3D R100 CBCT to screen for the presence of radiopaque mineralized deposits. Initial scans were performed with a resolution of 250 μm, followed by higher resolution scans (125 μm) in teeth suspected of having abnormal pulp structures/mineralization deposits. In all CBCT scans a tube voltage of 80 kV and a current of 8 mA were used.

### 2.3 High-resolution micro computed tomography - μCT

Teeth with CBCT indications for the presence of radiopaque mineralized deposits (*n* = 30, 25 central incisors and 5 lateral incisors) were further scanned non-destructively at high-resolution using an Easytom S system (RX solution, France) employing 80 kV, 116 μA (1 mm aluminum filter, 15 μm voxel size). Each tooth was scanned at multiple heights in a humidity-sealed container, clasped between water-saturated foam and polystyrene to maintain moisture and stability. Reconstructions (RX UniACT version 1.1) yielded high-definition 16-bit datasets, capturing the entire anatomy of each tooth (each sized 10–20 GB). For additional information see supplementary information document S1.

### 2.4 Laminography

Multiple polished slices of pulp stones (*n* = 17) were surface scanned by the Easytom S laminography configuration. This method makes it possible to probe internal scattering structures in wide, thin slices, with micrometer resolutions (Voropaev et al., 2016). For additional information see supplementary information document S2.

### 2.5 Optical imaging

**Fig. 1:**
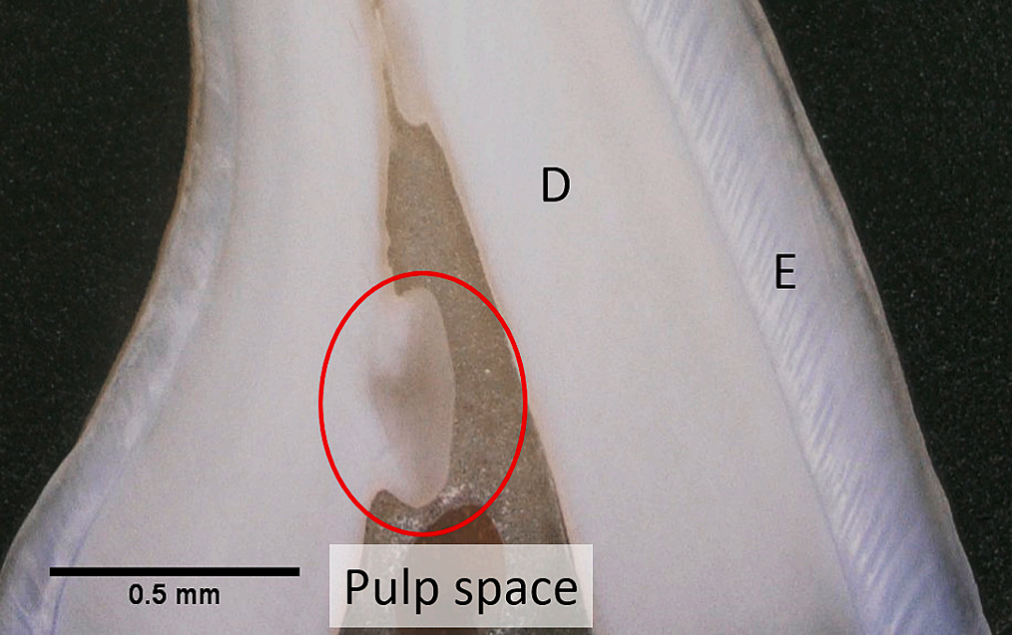
High-resolution optical microscopy image (Keyence) of a polished crown slice revealing a coronal pulp stone. The red circle highlights a stone that is adherent to the lingual wall of this pulp chamber. E: enamel, D: dentin. (For interpretation of the references to colour in this figure legend, the reader is referred to the web version of this article.)

All polished pulp stone slices were imaged with a Keyence microscope (VHX-S550E, KEYENCE CORPORATION, Japan) using a 200× objective, illuminated with the inbuilt coaxial light source, as shown in Fig. 1. The identified pulp stones in such sections were further analyzed by laminography and the methods described below.

### 2.6 Scanning electron microscopy - SEM

Polished pulp stone slices were imaged uncoated using a PhenomXL (Thermofisher Scientific, Netherlands), with the backscatter detector, 1000× magnification, 20 kV, 60 Pa low-vacuum mode. Several (*n* = 11) slices were also measured by EDX in a Phenom X Pro. For additional information about backscatter and EDX chemical imaging details, see supplementary information document S3.

### 2.7 Microfocus X-ray diffraction and fluorescence - XRD and XRF

One coronal and three radicular pulp stones (*n* = 4) selected to compare crown and root were measured at the mySpot microfocus beamline at the BESSY II synchrotron light source operated by the Helmholtz-Zentrum Berlin für Materialien und Energie (Berlin, Germany). XRD measurements were accompanied by XRF measurements. Roughly 1000 X-ray diffraction (XRD) patterns were collected in several maps across and along the exposed stones. Details about the experiment, data acquisition and processing are provided in the supplementary information document S4 and outlined graphically in supplementary Fig. S6. The raw X-ray diffraction patterns were analyzed using XRDUA software version 7.7 (De Nolf et al., 2014). A geometric calibration was first performed to refine the detector distance using Al_2_O_3_ as calibrant, followed by an azimuthal integration of the two-dimensional Debye-Scherrer rings. This procedure yielded one-dimensional intensity profiles for each scanned spot on the pulp stones. Each profile was subsequently fitted with a Voigt function in Origin Pro software (v10, OriginLab Corporation, USA) to determine the precise position of the main diffraction peak of the (002) reflection of carbonated hydroxyapatite (cHAP). The interplanar spacing (d-spacing) for each spot was calculated according to Bragg's law, and the crystallite size was estimated following the approach described by Forien et al. (Forien et al., 2016). The spatial distribution of these parameters was then visualized as colour maps using Fiji (ImageJ). The Zn K line peak in the XRF spectra was integrated to obtain the corresponding maps of the Zn distribution.

### 2.8 Low-vacuum laboratory micro-X-ray fluorescence spectroscopy - μ-XRF

To better identify the distribution of major and minor chemical elements inside the pulp stones, 10 individual pulp stones in seven slices from 4 teeth were analyzed. Micrometer-resolution laboratory μ-XRF measurements were conducted under low-vacuum (20 mbar) using a modified M4 Tornado μ-XRF spectrometer (Bruker Nano GmbH, Germany) (Lachmann et al., 2016) equipped with a rhodium X-ray tube within the joint research group SyncLab of the HZB at BLiX (Berlin Laboratory for innovative X-ray Technologies, TU Berlin, Germany). Lateral scans were used to create 2D maps of the main elements across entire tooth/stone slices. For additional details see the supplementary information document S5.

### 2.9 Data evaluation - μCT

The μCT scans of each tooth were reconstructed to create tomographic datasets capturing the full anatomy of each tooth. ImageJ/Fiji (Schindelin et al., 2015) was used to visualize and measure the dimensions of each identified stone as well as the surrounding canal diameter, to be able to determine stone volumes (one or several per tooth). Stone dimensions are presented as box plots, showing key statistics such as mean, median, and standard deviation. Only well-defined and morphologically distinct pulp stones were included. Normality was assessed with the Kolmogorov-Smirnov test. Statistical comparisons were made using Mann-Whitney *U* test applied for non-normally distributed data. Statistical significance was set at *p* < 0.05. All analyses were performed using Origin Pro (version 2022b, OriginLab Corporation, Northampton, MA, USA). μCT datasets were used to assess the spatial relationship between external wear and internal calcifications. Specifically, the remaining dentin thickness corresponding to the shortest distance from the attrition site to the pulp chamber was determined as well as the axial distance from the pulp chamber roof to the most coronal aspect of the first pulp stone. Prevalence, size distribution, and spatial characteristics of mineralized deposits were calculated per tooth. Comparisons between groups (e.g., attached vs free-standing deposits, coronal vs radicular locations) were performed using appropriate statistical tests, and significance was defined at p < 0.05.

## 3 Results

### 3.1 CBCT screening versus μCT imaging

Supplementary Video 1, Supplementary Video 2

The tomographic data helped us to non-destructively localize one or several stones in each of the 30 teeth. Fig. 3 and supplementary Fig. S2 provides example 3D renderings, close-ups and 2D views of typical coronal and radicular pulp stones, highlighting the lingual surface of the crowns where we observe signs of enamel attrition/erosion (blue arrows) due to tooth use. Note how that different teeth have different crown/root stone distributions: Fig. 3 (a) shows an example of a single coronal pulp stone, while Fig. 3 (b) displays multiple radicular pulp stones (identified with red arrows).

**Fig. 2:**
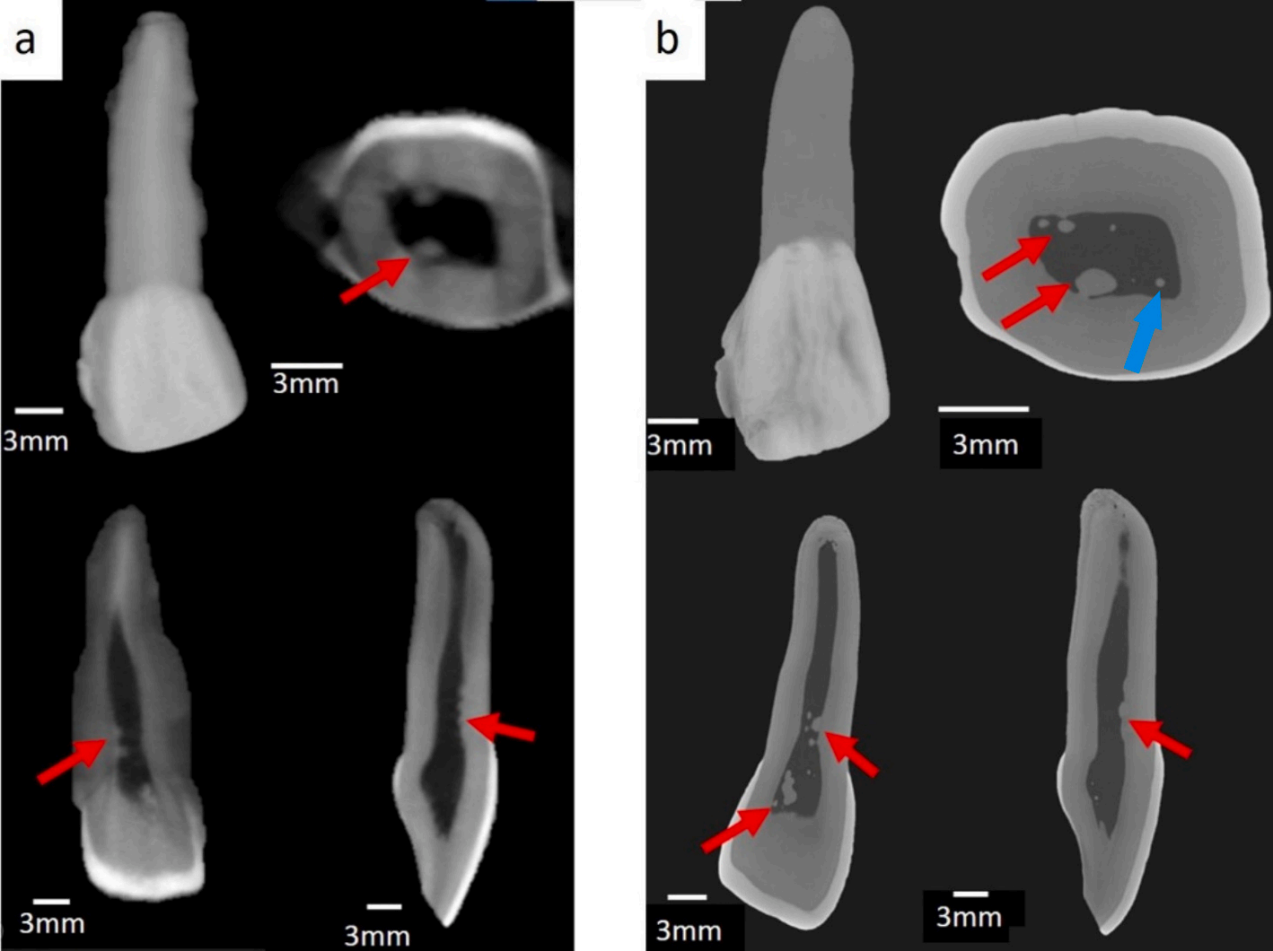
(a) Example CBCT image of a bovine tooth displaying a pulp stone. (b) Corresponding μCT image of the same tooth, with pulp stones indicated by red (attached) and blue (free standing) arrows. (For interpretation of the references to colour in this figure legend, the reader is referred to the web version of this article.)

**Fig. 3:**
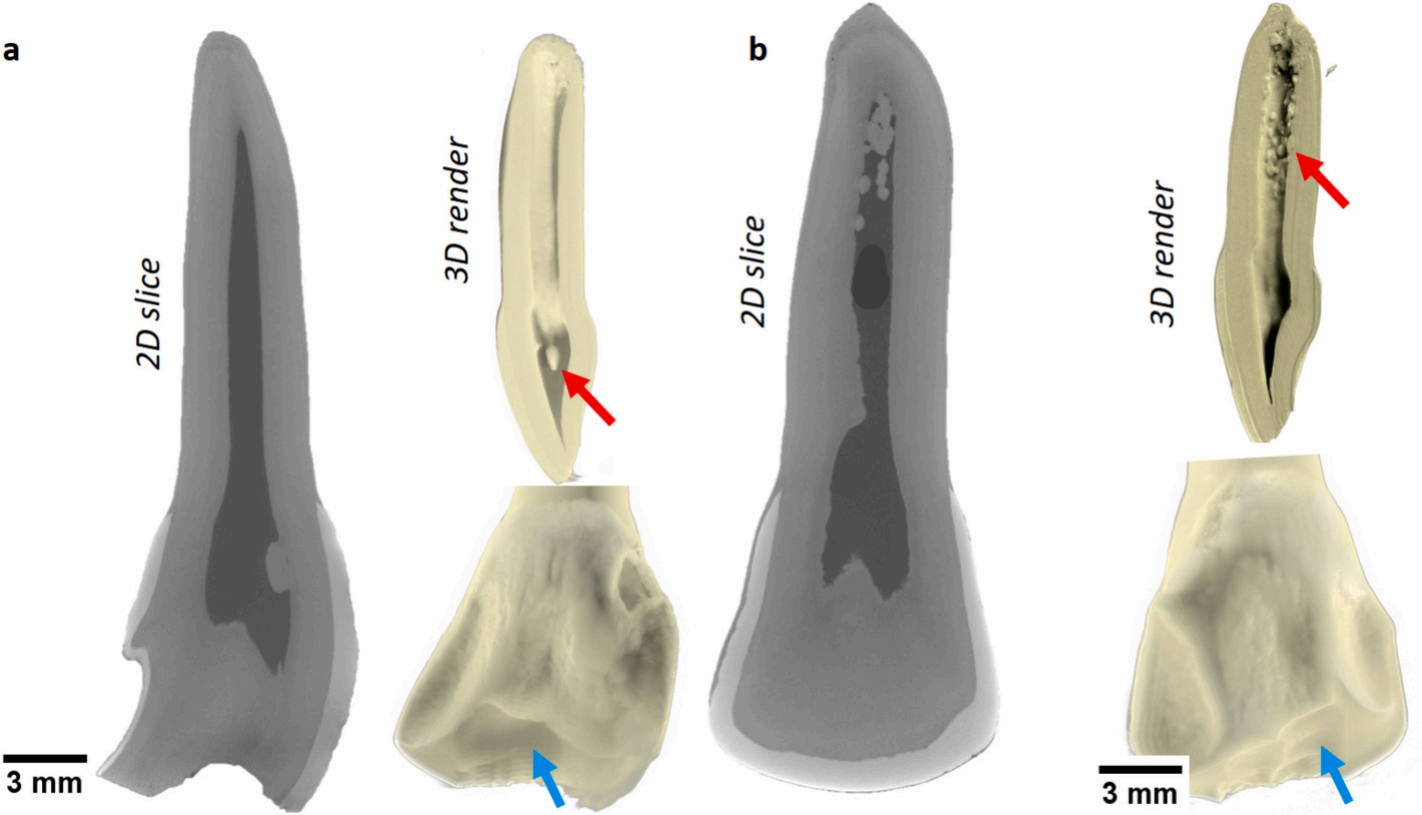
Typical 3D µCT renderings of two bovine teeth highlighting the presence of pulp stones (red arrows). (a) A tooth with a coronal pulp stone. (b) A tooth with radicular pulp stones. Red arrows indicate the location of the pulp stones in digital virtual cuts in the slices, while blue arrows point to wear and enamel attrition on the outer lingual surface of the crown. (For interpretation of the references to colour in this figure legend, the reader is referred to the web version of this article.)

### 3.2 Tomographic high-resolution 3D size distribution of pulp stones

**Fig. 4:**
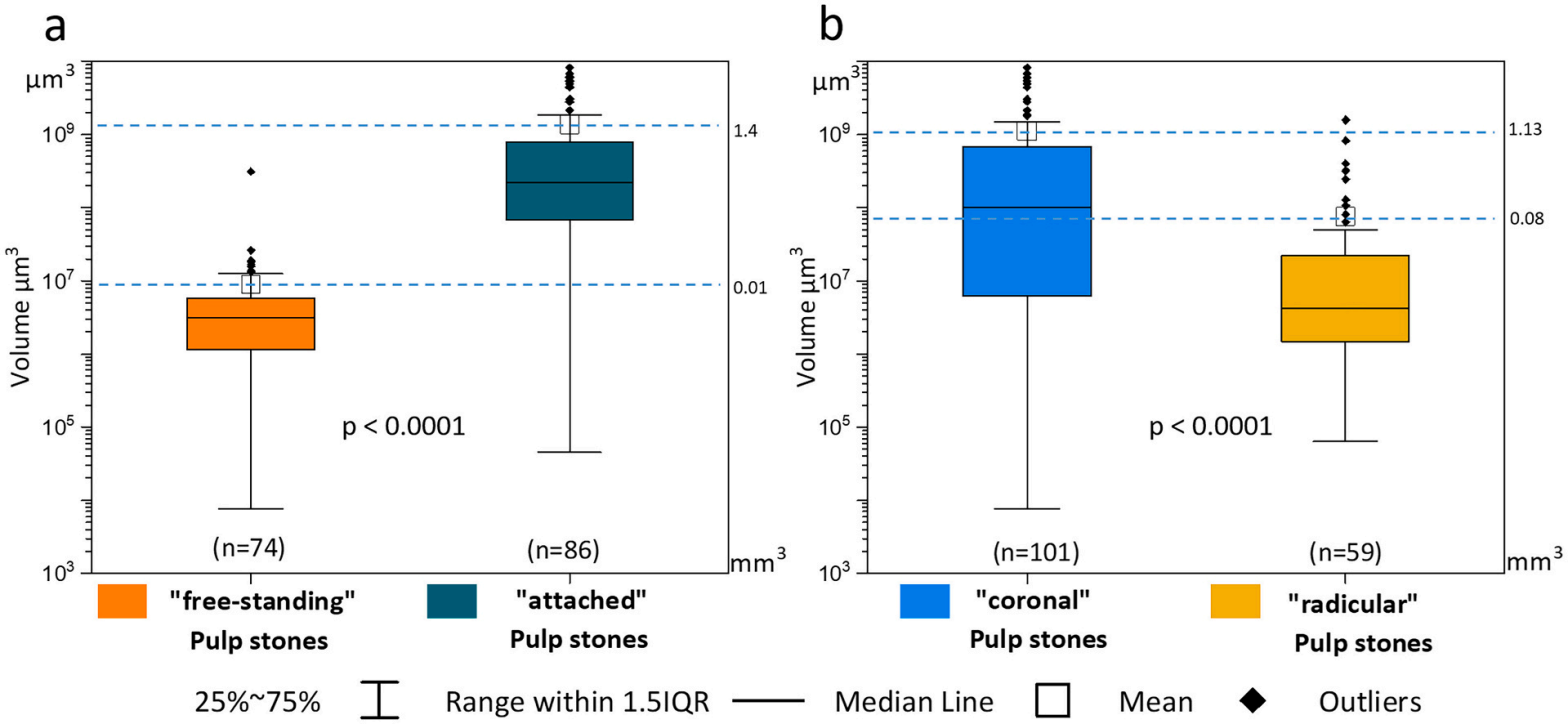
Box plot illustrating the volumes of pulp stones (*n* = 160) reported in μm^3^. (a) Compares the volumes of “free-standing” (n = 74) and “attached” (n = 86) pulp stones. (b) Contrasts the same stones as volumes of coronal (n = 101) versus radicular (n = 59). The plot highlights the distribution and variability of pulp stone volumes. Note that, due to the large range of sizes, a logarithmic scale is used.

A total of 160 mineralized deposits were identified as pulp stones and grouped according to (i) anatomical location—coronal (*n* = 101) and radicular (*n* = 59)—and (ii) morphological classification—attached (*n* = 86) and free-standing (*n* = 74). Volume distributions are presented in Fig. 4 as box plots, including mean, median, interquartile range (IQR), standard deviation, and outliers. Overall, deposit volumes spanned approximately five orders of magnitude. Free-standing deposits were smaller, with a narrow IQR and low median values. In contrast, attached deposits exhibited larger volumes and greater variability, including several high-volume outliers. In some cases, accurate volumetric delineation of attached deposits was limited by partial embedding within dentin walls.,

When stratified by anatomical location (Fig. 4b), coronal deposits were larger on average and displayed a wider IQR compared to radicular deposits, which showed smaller volumes and a more uniform distribution. Mean volumetric values further illustrate these differences: coronal deposits (*n* = 101) had an average volume of 1.13 ± 2.04 mm^3^, whereas radicular deposits (*n* = 59) averaged 0.08 ± 0.20 mm^3^. The attached deposits (*n* = 86) were larger (mean: 1.4 ± 3.7 mm^3^) compared to free-standing deposits (*n* = 74), which averaged 0.01 ± 0.04 mm^3^.

A Kolmogorov-Smirnov test indicated that pulp stone volumes are not normally distributed across the two groupings (coronal, versus radicular, “free-standing”, versus “attached”). Accordingly, non-parametric comparisons were performed using the Mann–Whitney *U* test, with statistical significance set at *p* < 0.05. Statistical analysis confirmed that attached deposits exhibited significantly larger volumes than free-standing deposits, based on both mean and median values. Similarly, coronal deposits were substantially larger than radicular deposits, with mean volumes approximately 14-fold higher. Supplementary table S1 provides the distribution and attributes of the different stones, showing predominately radicular stones in middle/apical thirds (28 and 28 stones respectively), with minimal presence near the tooth cervical region. A positive correlation (r ≈ 0.9) was observed between the diameter of attached deposits and the diameter of the surrounding root canal (Supplementary Fig. S3). Analysis showed that in these cases, attached deposits occupied approximately one-third of the canal diameter.

Morphometric analysis based on μCT data (Supplementary Table S2) quantified both the remaining dentin thickness in regions of attrition and the axial distance from the pulp roof to the first detected deposit. No correlation was observed between remaining dentin thickness and the axial position of the uppermost detected mineralized deposit.

### 3.3 Laminography versus μCT

Supplementary Video 3, Supplementary Video 4.

### 3.4 Optical versus electron microscopy

High-resolution imaging by optical microscopy and backscattered electron scanning electron microscopy (BSE–SEM) revealed structural and contrast differences clearly distinguishing between mineralized deposits and the surrounding dentin.

**Fig. 5:**
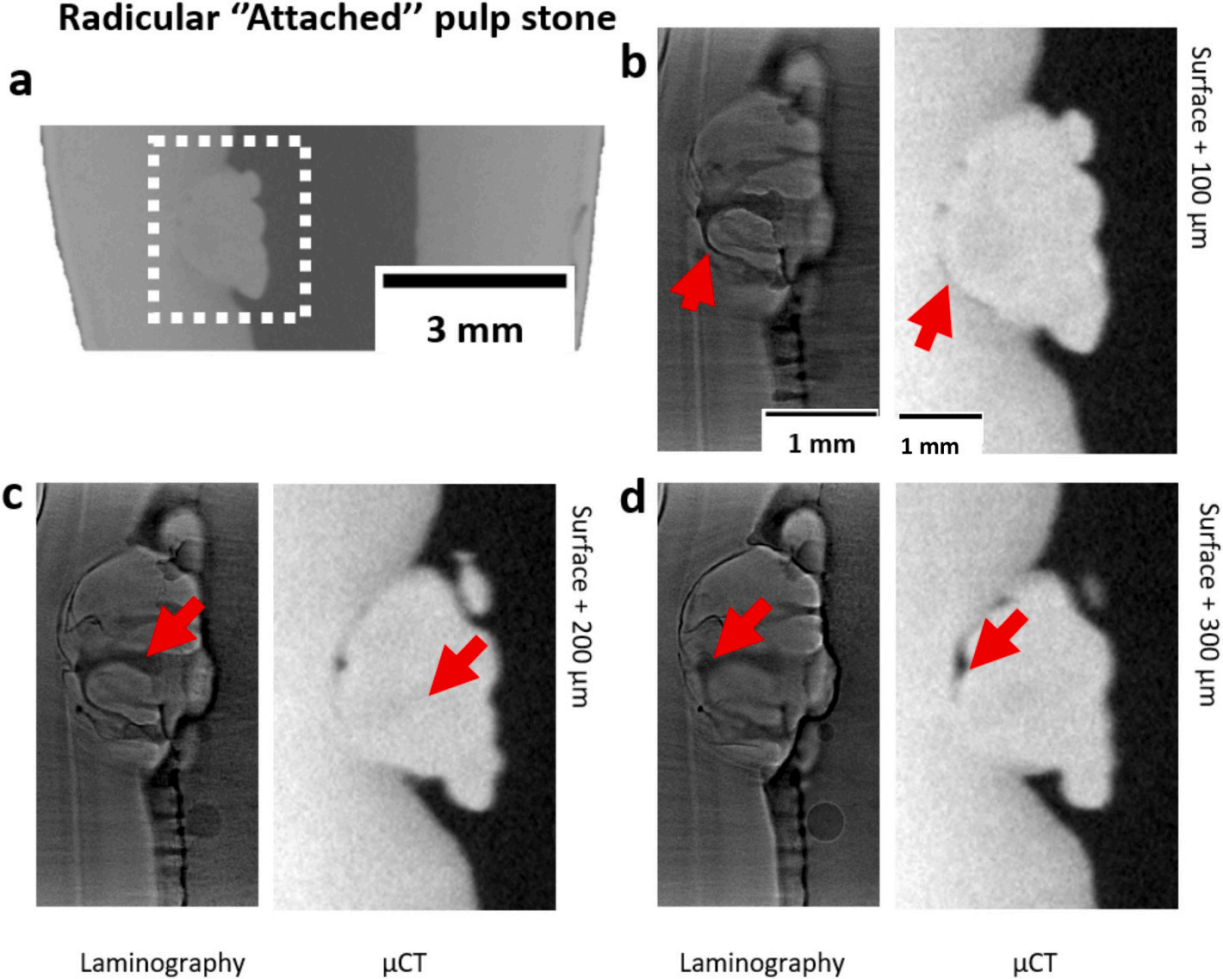
μCT versus laminography in a sliced radicular pulp stone. (a) Longitudinal section of a μCT scan of a root showing a radicular pulp stone. (b) Images at 100 μm depth beneath the surface, where the red arrow highlights a small radiolucent structure. (c) Images at 200 μm depth, indicating a small radiolucent structure that could represent an internal canal. (d) Images at 300 μm depth, the red arrow points to a small radiolucent feature that may be a pore. Laminography thus allows for the visualization of these fine internal details, which are not usually discernible by μCT. 1 mm scale valid for all shown slices.

**Fig. 6:**
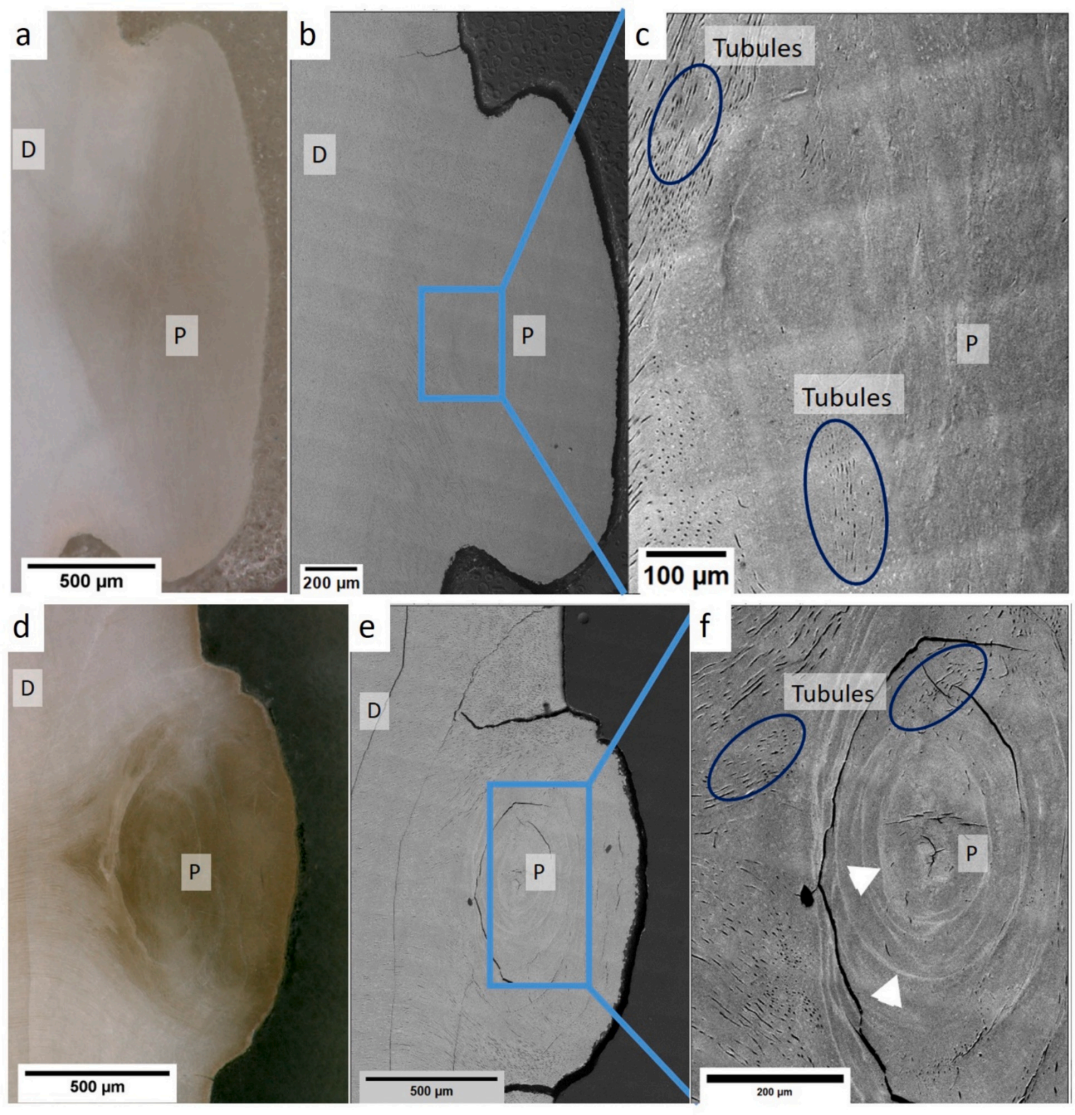
(a) Light microscope image of a longitudinal section of an “attached” coronal pulp stone, displaying a distinct colour contrast compared to normal dentin. (b) BSE-SEM image of the same coronal pulp stone imaged in low vacuum (60 Pa), revealing a heterogeneous internal structure with density variations appearing as alternating dark and light regions. The brighter areas, characterized by a higher electron backscatter signal, suggest a composition of heavier elements or greater mineral density, while the darker regions in (a) indicate reduced optical scatter, suggesting fewer or no tubules. Tubular structures do appear sparsely in some regions. Concentric structures, successive layers of varying electron densities, are often evident. (c) An inset showing both normal dentin and the coronal pulp stone in more detail under magnification. The oblique white lines visible in the image are artifacts resulting from image stitching, not actual pulp-stone structural features. (d) Light microscope image of a longitudinal section of a radicular pulp stone, showing a colour difference compared to normal dentin. (e) BSE-SEM image of the same radicular pulp stone, illustrating texture heterogeneity, with alternating bright and dark regions indicating varying composition or density. (f) An inset showing both normal dentin and the radicular pulp stone in more detail under magnification. Insets for both coronal and radicular pulp stones provide closer views,. D: dentin, P: pulp stone, arrowheads point to the mineral layers, blue oval: dentinal tubules. (For interpretation of the references to colour in this figure legend, the reader is referred to the web version of this article.)

Optical microscopy (Fig. 1) at high magnification (Fig. 6a) of a longitudinal section of a coronal attached pulp stone showcases a distinct colour contrast between the deposit and adjacent dentin. The corresponding BSE–SEM image (Fig. 6b) appears relatively homogeneous at lower magnification, while higher magnification imaging (Fig. 6c) revealed localized internal features, including layered structures and occasional tubule-containing domains.

Similarly, longitudinal sections of radicular attached deposits exhibited visible contrast differences relative to surrounding dentin under optical microscopy. Corresponding BSE–SEM images (Fig. 6e–f) demonstrated multiple concentric layers with distinguishable variations in backscattered electron contrast. Note that imaging artifacts such as cracks and faint linear features were observed in some specimens. These are attributable to sample damage due to mechanical polishing or dehydration as well as localized electron charging during SEM analysis, which are not present in the 3D X-ray measurements. In total, 14 attached deposits exhibited identifiable tubule-like features, distributed equally between coronal (*n* = 7) and radicular (n = 7) regions.

### 3.5 SEM-EDX elemental analysis

Complementary spectroscopic analyses were performed on a subset of exposed pulp stones (*n* = 11) to assess their elemental composition. SEM–EDX was used for localized elemental composition analysis of regions exhibiting different contrast levels of micron sized domains in the backscattered electron (BSE) imaging. In such images, regions of higher intensity correspond to increased electron backscatter, while lower-intensity regions exhibit reduced signal (Supplementary Fig. S5). EDX spectra obtained from coronal stones (Supplementary Fig. S5a) showed that regions of higher BSE intensity contained Ca and P levels comparable to adjacent dentin. Regions of lower BSE intensity exhibited similar elemental profiles, with relatively lower signal intensities for these elements. Radicular stones (Supplementary Fig. S5b) displayed comparable elemental compositions across analyzed regions, with visible contrast differences corresponding to layered structural features. Across all analyzed specimens, the dominant elements detected were Ca, P, O, and *C.* M*inor* elements, including Mg, Na, and Zn, were also detected with variable but overall, very low signal intensities. No major elemental compositional differences were identified that could explain the contrast variations observed in BSE imaging suggesting that it is mainly the density and not the composition, that varies.

### 3.6 Correlations between SEM, X-ray transmission, XRD and XRF

In a subset of specimens, including 1 coronal and 3 radicular deposits, it was possible to directly compare SEM, XRD, and XRF measurements on the same stones.

**Fig. 7:**
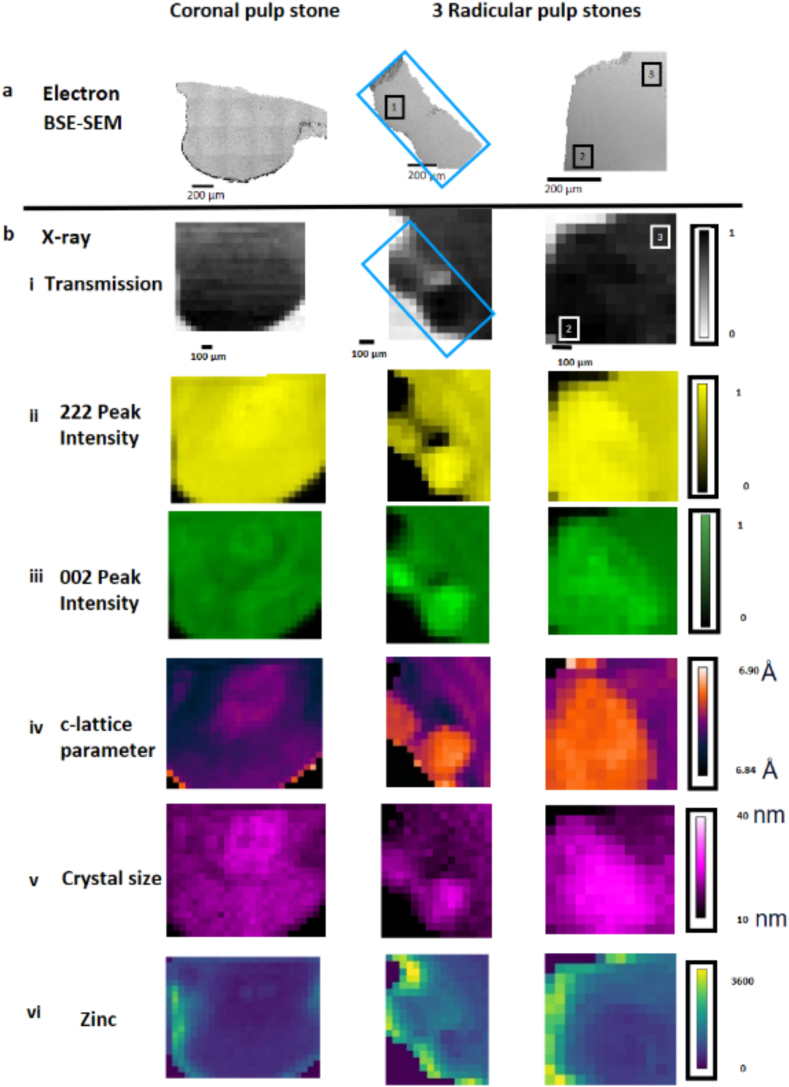
Structural, elemental and crystallographic maps example of coronal and radicular pulp stones. (a) BSE-SEM images provide an overview of the analyzed stones. (b) A series of maps for the same stones: (i) X-ray transmission showing subtle internal density variations, (ii) integrated intensity of the apatite (222) reflection, (iii) integrated intensity of the apatite (002) reflection, (iv) calculated c-lattice parameter, (v) apatite crystallite size, and (vi) Zn K fluorescence intensity. The left column represents a coronal stone, while the middle and right columns show two different radicular stones. Note that zinc is predominantly localized at the peripheries and is not spatially correlated with variations in the c-lattice parameter, particularly in the radicular stones where the c-lattice parameter is visibly larger. Colour bars indicate the relative signal intensity for each map. For points 1, 2, and 3 we calculated the mineral density based on transmission/absorption which yielded values of 1.44 g/cc, 1.48 g/cc, and 1.33 g/cc, respectively.

At sub-micrometer resolution, BSE–SEM imaging revealed relatively uniform contrast on the polished surfaces, comparable to adjacent dentin (Fig. 7a). In contrast, X-ray transmission mapping (Fig. 7b), which probes the full thickness of the slice, revealed internal variations in absorption, including localized regions of increased attenuation not apparent in the corresponding SEM surface imaging. Density estimates derived from X-ray transmission measurements at selected locations (points #1–#3) ranged from 1.33 to 1.48 g/cm^3^. These values were lower than typical dentin reference values (∼2.0 g/cm^3^), indicating differences in bulk density XRD measurements acquired at the same locations identified apatite as the dominant crystalline phase in both pulp stones and adjacent dentin. Subtle variations in the intensity of the 002 and 222 diffraction features of the apatite crystals were observed across different regions, suggestive of regional variations in crystallinity. The 002 peak corresponds to the c lattice parameter of apatite (the typical carbonated apatite of teeth and bones) (Forien et al., 2016; Forien et al., 2015b; Mills, 2014). The calculated c-lattice parameter showed small spatial variability: in the analyzed coronal deposit, values were comparable to those of adjacent dentin, whereas radicular deposits exhibited higher c-lattice parameter values compared to adjacent dentin. The estimated crystallite size was aslo similar to normal dentin and ranged from approximately 20 to 30 nm (Fig. 7). XRF spectra collected at the same locations (Supplementary Fig. S6) detected Ca and P as major elements, along with localized Zn enrichment in certain regions. The spatial distribution of Zn did not show a consistent association with variations in c-lattice parameters or crystallite size in the analyzed specimens (Mantouvalou et al., 2026).

**Fig. 8:**
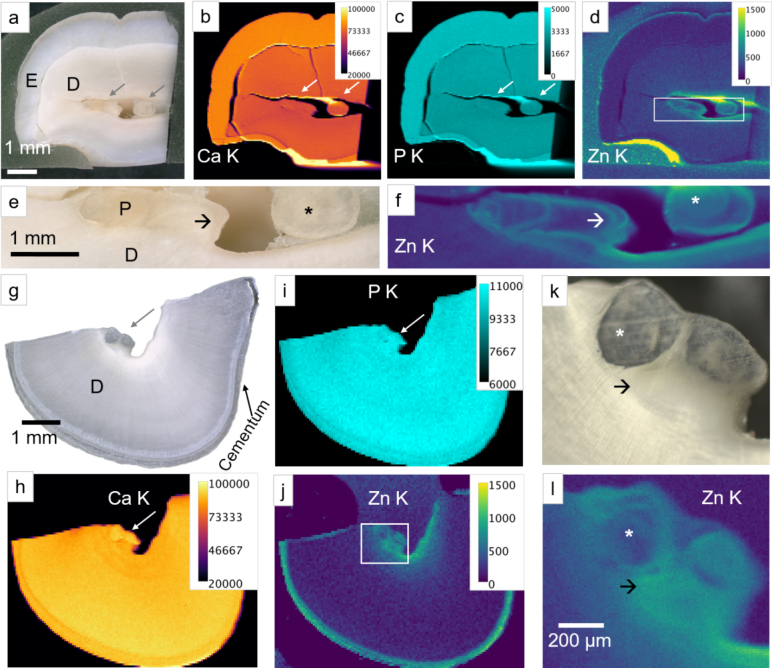
X-ray fluorescence imaging of bovine tooth cross-sectional slices exposing coronal and radicular pulp stones. (a) An optical image of half of a cross-sectional slice from a bovine tooth. Matching arrows in figures (a–c) indicate the location of the coronal pulp stones. The scale bar in (a) applies to figures (a–d); (b–d) Overview μ-XRF maps of Ca K, P K, Zn K fluorescence measured with a 50 μm pixel size, 1 s per pixel, collected under 20 mbar vacuum. The white rectangle in (d) in the middle marks the measurement location shown at higher resolution in panels (e–f); (e) Close-up optical image of the pulp coronal stones; (f) Zn K μ-XRF map of a magnified region with pulp stones, measured with a 10 μm pixel size, 3 s per pixel, and 20 mbar vacuum. (g) Optical image of a cross-sectional slice from a bovine tooth with radicular pulp stones. The arrows in figures (g–i) indicate the location of two radicular pulp stones. The scale bar in figure (g) applies to figures (g–j). (h–j) Overview μ-XRF maps of the Ca K, P K, and Zn K fluorescence measured with a 50 μm pixel size, 1 s per pixel, 2 mbar vacuum, and aluminum 12.5 μm filter. The white rectangle in figure (j) identifies a region mapped at higher resolution as shown in figures (k-I); (k) Higher magnification optical image of the pulp stones in (g); (l) Zn K μ-XRF map of a detailed region with pulp stones, measured with a 10 μm pixel size, 3 s per pixel, and 2 mbar vacuum. An asterisk (*) marks the pulp stone, while the black arrow highlights an area exhibiting a layered structure, suggesting variations in mineral deposition and potential differences in the developmental history. Note the difference in composition as compared with the surrounding dentin matrix. E: enamel, D: dentin, P: pulp stone. The XRF intensities are given in counts per second (cps).

**Fig. 9:**
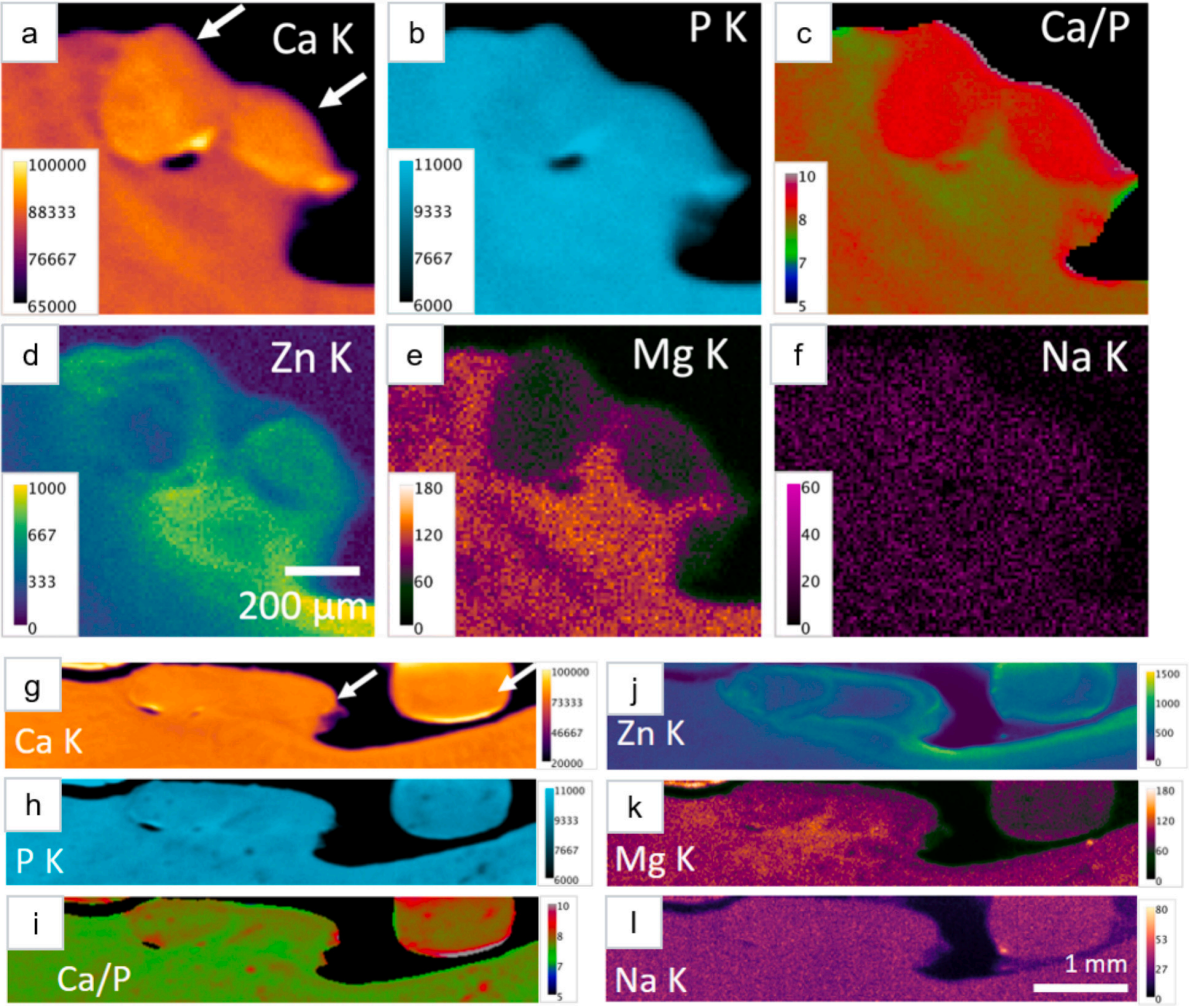
X-ray fluorescence imaging of radicular and coronal pulp stones in a bovine tooth slice. (a–b, d–f) Detailed μ-XRF maps of the Ca K, P K, Zn K, Mg K, and Na K fluorescence of radicular pulp stone measured with a 10 μm pixel size, 3 s per pixel, and 20 mbar vacuum. The arrows in (a) indicate the location of two pulp stones. The scale bar in (d) applies to figures (a-e). (c) A relative Ca/P ratio map was generated by pixel-wise division of deconvolved Ca K and P K net-intensity maps (ImageJ). (g–h, j–l) Detailed μ-XRF maps of the Ca K, P K, Zn K, Mg K, and Na K fluorescence of coronal pulp stone measured with a 10 μm pixel size, 3 s per pixel, and 2 mbar vacuum. The arrows in (g) indicate the location of two pulp stones. (i) A Ca/P ratio map was generated by pixel-wise division of deconvolved Ca K and P K net-intensity maps (ImageJ). The scale bar in (l) applies to (g-k). The intensity scale in all panels represents the net peak intensity in cps. Low-signal P and Ca pixels were masked to avoid artifacts from division by near-zero intensities.

Elemental mapping using μ-XRF under vacuum conditions provided additional insights regarding the spatial distribution of major and minor elements within the samples (Fig. 8). Ca and P exhibited predominantly homogeneous distributions in dentin, with localized variations observed near the periphery of the pulp stones. Within the analyzed deposits, Ca and P signals were uniformly distributed, with only minor spatial fluctuations visible. Zn K-line signals, originating from depths of up to approximately 100 μm below the exposed surface, showed heterogeneous spatial distributions within both coronal and radicular deposits (Fig. 8d, f, j, l). In several specimens, Zn signals appeared in layered or band-like patterns. Compared to adjacent dentin (Mantouvalou et al., 2026), increased Zn signal intensities were observed in multiple pulp stone regions. Representative examples are shown in Fig. 9. High-resolution μ-XRF maps further illustrate the spatial distribution of Ca, P, Zn, Mg, and Na, as well as localized variations in Ca/P ratios within the analyzed specimens. No consistent spatial relationship was observed between Zn distribution and structural features identified by other imaging modalities.

## 4 Discussion

This study reports on the chance finding of multiple pulp stones in a large cohort of extracted seemingly-intact Bavarian bovine teeth taken from the meat industry. The data presented integrates multi-modal materials-science approaches to characterize pulp-associated mineralizations found in a large proportion (30%) of young bovine incisors subjected to limited functional use (≤2 years). All teeth were obtained from 3-4 year-old cattle without caries or prior dental treatment, providing a model system relatively free from age- and pathology-related confounders. While we cannot be sure that each tooth tested originated in a different animal, the random sampling of 100 teeth from within ca. 1000 extracted teeth suggests that about 30 % of visibly intact bovine teeth contain radiopaque mineralized deposits within the dentin–pulp complex, despite otherwise normal macroscopic appearance. These findings indicate that ectopic mineralization is relatively common even in young, clinically untreated teeth and is not likely to be an aging process.

The μCT-based analysis represents the most elaborate component of this study. It enabled comprehensive three-dimensional assessment of prevalence, spatial distribution, and morphometric characteristics across the full sample set. Mineralized deposits were observed both as free-standing structures within the pulp tissue and also as masses attached to canal walls (Fig. 4). Attached deposits were significantly larger than free-standing ones, and substantial variability in size and location was evident. These findings are consistent with previous reports in human teeth (Goga et al., 2008), although the present study identified a predominance of deposits in the apical and middle thirds rather than the cervical region, and we are certain that there was no dental treatment nor caries associated with our chronologically-young samples. Indeed, the presence of multiple stones within a single tooth (*n* = 26) and the lack of a consistent relationship between coronal and radicular occurrences suggest that pulp stone formation is not governed by a single localized factor.

While the μCT findings provide statistically supported insights, the extremely resource-intensive multimodal characterization (laminography, SEM–EDX, μ-XRF, and XRD) was conducted on a limited subset of specimens and should therefore be interpreted as exploratory. Within these analyzed slices, heterogeneous internal architectures were observed, including layered structures and channel-like features. Such patterns are consistent with previously described mineralization mechanisms, including incremental deposition and coalescence processes (Berès et al., 2016; Demant et al., 2022; Deva et al., 2006; Moss-Salentijn and Klyvert, 1983). This attests to the relevance of the young bovine-incisor pulp stones as a possible model for pulp-stone research in future studies.

Density variations observed within the mineralized bodies likely reflects differences in mineral–organic ratios. Note that our results are completely consistent with other studies reported in aged human teeth (He et al., 2022). Similarly, tubule-like structures were occasionally observed in attached stones, suggesting possible interactions with dentin-forming cells, while other regions appeared structurally disorganized. Such heterogeneity has also been reported in human pulp stones (Berès et al., 2016; Johnson and Bevelander, 1956; Le May and Kaqueler, 1991; Milcent et al., 2019), supporting the notion that these mineralizations arise through complex and potentially variable biological pathways which are similar across mammals.

XRD analysis confirmed hydroxyapatite as the dominant crystalline phase in the few samples that we mapped, consistent with previous reports in human teeth (Aoba et al., 1980; Berès et al., 2016; Xu and Wang, 2012) but different to a previous report in a single bovine tooth (Kodaka et al., 1998). Subtle variations in crystallographic parameters between different regions and between pulp stones and adjacent dentin were observed, which may reflect differences in mineral maturity, organic matrix interactions, or local physicochemical stress conditions (Forien et al., 2016; Forien et al., 2015a; Schemenz et al., 2020). Further work is needed to better understand and fully characterize these observations.

Zinc was consistently detected in all examined specimens, with localized enrichment observed primarily at peripheral regions of the mineralized deposits. Similar spatial patterns have been reported in both human pulp stones and other mineralized tissues (Berès et al., 2016; Milcent et al., 2019). While zinc has been associated with enzymatic activity, matrix stabilization, and antimicrobial responses (McCall et al., 2000; Molenda and Kolmas, 2023; von Pein et al., 2021), the present study does not provide direct evidence for any specific biological functional role. The observed distribution may reflect localized mineralization dynamics, but further in-vivo dedicated investigations are required to fully establish this.

From a functional perspective, all examined teeth exhibited signs of attrition, consistent with the natural use of bovine incisors. Mechanical loading (Fleck et al., 2020) and exposure of dentinal tubules may contribute to changes in the dentin–pulp environment. However, although the distribution and morphology of the observed mineralizations are compatible with such influences, the present study was not designed to establish causal relationships. Similarly, while microbial involvement remains biologically plausible (Farges et al., 2003; Sedgley, 2017; Siqueira et al., 2010), no direct evidence is possible in such an ex-vivo study.

The bovine incisor model offers several advantages for studying early-stage mineralization, including controlled age range, absence of clinical interventions, and structural similarity to human dentin (de Dios Teruel et al., 2015). At the same time, species-specific differences must be considered when extrapolating findings to human biology. The relatively high prevalence of mineralized deposits observed in this study suggests that bovine incisors may provide a useful experimental system for investigating early mineralization processes under controlled conditions.

### 4.1 Limitations

This study has several limitations. First, we cannot rule out that multiple teeth may originate from the same animal, and therefore potential within-animal clustering may affect statistical independence of the observations, particularly for prevalence and morphometric analyses, though each tooth represents its own pulp-stone development environment. Second, advanced characterization techniques (laminography, SEM–EDX, μ-XRF, and XRD) were applied only to a limited subset of specimens due to the extensive costs and complexity and should therefore be interpreted as indicative rather than definitive. Still they add to the results obtained in other studies complementing the overall scientific picture. An additional limitation is that the cross-sectional design and lack of longitudinal data limit conclusions regarding the temporal progression of pulp stone formation. We do know that the pulp stones appear after partial or complete development of the incisors and that the animal age at slaughter is such that the teeth were used for no more than 3 years on regular cattle feed. Additionally, the ex-vivo study design and absence of viable pulp tissue precluded direct biological or cellular analyses. Finally, while bovine teeth provide a useful model system, species-specific differences may limit direct extrapolation to human conditions.

## 5 Conclusions

Within the limitations of this study, pulp-associated mineralizations were frequently observed in young bovine incisors. These pulp stones exhibited substantial variability in size, location, and structure. μCT-based analyses provide evidence of a relatively high prevalence manifesting some morphometric diversity, while multimodal characterization suggests structural and chemical heterogeneity at higher micrometer, nanometer and atomic length scales. These findings make it interesting to consider the use of bovine teeth as a complementary model for investigating early-stage mineralization processes in the dentin–pulp complex. Further studies integrating biological, chemical, and longitudinal approaches are required to clarify the mechanisms underlying pulp stone initiation and development.

The following are the supplementary data related to this article.

Supplementary Video 1 Three-dimensional visualization of a bovine incisor using a high-resolution (0.125 mm voxels) clinical cone-beam computed tomography (CBCT) system, highlighting the limited spatial resolution and reduced ability to detect fine details within the pulp chamber.

Supplementary Video 2 High-resolution micro-computed tomography (μCT) visualization of the same bovine incisor shown in Video 1. The scan at 15 µm resolution reveals significantly more anatomical detail, clearly delineating pulp stones and internal features within the pulp chamber.

Supplementary Video 3 Laminographic visualization of increasing depths within a cut and polished coronal pulp stone. Cross-sectional slices reveal concentric internal layering and subtle mineral density variations that are not detectable by standard μCT, indicating structural heterogeneity and potential remnants of organic matrix.

Supplementary Video 4 Laminographic visualization of a caut and polished radicular pulp stone (compare Supplementary video 3). The video reveals pronounced internal channel-like structures and alternating high- and low-density domains, supporting the interpretation of non-uniform mineralization and possible variable organic inclusions.

Supplementary material

## CRediT authorship contribution statement

**Hamza Elfarraj:** Writing – review & editing, Writing – original draft, Visualization, Validation, Software, Methodology, Investigation, Data curation. **Oleksandra Marushchenko:** Writing – review & editing, Writing – original draft, Visualization, Software, Investigation, Formal analysis, Data curation. **Franco Lizzi:** Writing – review & editing, Writing – original draft, Visualization, Validation, Software, Investigation, Formal analysis, Data curation. **Leona J. Bauer:** Writing – review & editing. **Ivo Zizak:** Visualization, Validation, Supervision, Resources, Methodology. **Hagay Shemesh:** Writing – review & editing, Writing – original draft, Supervision, Methodology, Investigation, Conceptualization. **Ioanna Mantouvalou:** Writing – review & editing, Writing – original draft, Validation, Supervision, Resources, Methodology, Investigation, Funding acquisition, Data curation, Conceptualization. **Paul Zaslansky:** Writing – review & editing, Writing – original draft, Visualization, Validation, Supervision, Software, Resources, Methodology, Investigation, Funding acquisition, Conceptualization.

## Consent for publication

Consent for publishing has been obtained from all authors. All authors read and approved the manuscript.

## Ethical statement

This article does not contain any studies with human participants or animals performed by any of the authors.

## Declaration of generative AI and AI-assisted technologies in the writing process

During the preparation of this work the authors used Grammarly in order to check syntax and shorten the text. After using this tool/service, the authors reviewed and edited the content as needed and take full responsibility for the content of the publication.

## Funding

The study was funded by DAAD (German Academic Exchange Service) and the DFG (German Research Foundation).

## Declaration of competing interest

The authors declare that they have no known competing financial interests or personal relationships that could have appeared to influence the work reported in this paper.

## Data Availability

Data will be made available on request.
