# Supplementary material for: Structural and chemical heterogeneity of pulp stones in young untreated bovine incisors: frequent dentin-like mineralizations
Source: J Struct Biol X. 2026 Jul 6;14:100151. doi: 10.1016/j.yjsbx.2026.100151 (PMC13446348; doi:10.1016/j.yjsbx.2026.100151)
Supplement: Supplementary file 1 [file mmc5.docx]

**Structural and chemical heterogeneity of pulp stones in young untreated bovine teeth: neither hyper nor hypo mineralization frequently appearing in the dentin-pulp complex**

**Supplementary Information**

**S1. High-Resolution 3D Tomographic Imaging of Pulp Stones: Comparing Size Distributions from CBCT Screening and µCT**

To provide a detailed overview of the spatial distribution of pulp chamber biominerals, we quantified their occurrence within coronal and radicular regions of bovine incisors. Deposits were further categorized as free (discrete within the pulp space) or attached (anchored to dentin walls). The data in Supplementary Table S1 highlight that the majority of stones were found in the coronal pulp chamber, while radicular stones were predominantly observed in the middle and apical thirds. To clarify the terminology used for regional classification in supplementary table S1, Fig. S2 presents a CBCT-based 3D rendering of a bovine central incisor illustrating the anatomical subdivisions of the pulp cavity. These include the roof, coronal, middle, and cervical thirds of the pulp chamber, as well as the coronal, middle, and apical thirds of the root canal.

**Fig. S1.** 3-D rendering of a bovine central incisor obtained using cone-beam computed tomography (CBCT), illustrating the anatomical subdivisions of the pulp cavity applied for stone localization analysis. The image delineates the roof, coronal, middle, and cervical thirds of the pulp chamber, as well as the coronal, middle, and apical thirds of the root canal. Labeled brackets and arrows indicate the terminology used for regional classification in Supplementary Table S1.

An additional group of pulp stones found were those observed embedded within dentinal walls. This suggests that some pulp stones in bovine teeth initiate early during tooth development, possibly even before eruption.

**Fig. S2.** Typical 3D renderings of attached and free pulp stones.

**Fig. S3.** (a) Correlation analysis between root canal diameter (up to 5,500 µm) and pulp stone diameter for ‘’attached ‘’ pulp stones. A strong positive correlation (r = 0.9) was observed within the shaded region for ‘’attached ‘’ pulp stones, with a linear fit equation of y = 0.3x -132, indicating that the diameter of ‘’attached ‘’ pulp stones is approximately one-third the size of the root canal diameter. However, this equation is only valid within the shaded area, as several outliers with larger canal diameters deviate from this trend. (b)” free-standing” pulp stone. (c) Coronal pulp stone. (d) Radicular pulp stone.

**Supplementary Table S1.** Distribution of pulp stones in bovine incisors according to anatomical location and attachment status. Values represent the number of free and attached stones, total counts, and prevalence (%) within coronal and radicular regions.

| Location | Free Stones (n) | Attached Stones (n) | Total Stones (n) | Prevalence (%) |
| --- | --- | --- | --- | --- |
| **Coronal** | 32 | 69 | 101 | 63% |
| - Roof | 0 | 14 | 14 |  |
| - Middle | 32 | 44 | 76 |  |
| - Cervical extension | 1 | 11 | 12 |  |
| **Radicular** | 37 | 22 | 59 | 37% |
| - Cervical | 0 | 3 | 3 |  |
| - Middle | 16 | 12 | 28 |  |
| - Apical | 21 | 7 | 28 |  |

**Supplementary Table S2.** qualitative attrition grading and associated enamel and pulp proximity measurements.

| Sample | Distance from attrition area to pulp (mm) | Distance from pulp roof to 1st stone (mm) |
| --- | --- | --- |
| 1 | 8.98 | 0 |
| 2 | 6.96 | 0 |
| 3 | 3.99 | 3.54 |
| 4 | 8.95 | 0 |
| 5 | 8.56 | 0 |
| 6 | 0.98 | 3.81 |
| 7 | 7.31 | 0 |
| 8 | 5.63 | 0 |
| 9 | 4.74 | 0 |
| 11 | 7.99 | 0 |
| 12 | 4.59 | 4.95 |
| 13 | 4.27 | 5.15 |
| 14 | 5.74 | 0 |
| 15 | 5.29 | 3.47 |
| 17 | 5.50 | 0 |
| 18 | 3.28 | 0 |
| 19 | 2.76 | 0 |
| 20 | 8.01 | 0 |
| 21 | 7.88 | 0 |
| 22 | 5.53 | 0 |
| 23 | 6.74 | 0 |
| 24 | 8.97 | 0 |
| 25 | 3.49 | 0 |
| 26 | 6.20 | 0 |
| 27 | 6.00 | 0 |
| 28 | 8.82 | 0 |
| 29 | 4.55 | 0 |
| 30 | 6.24 | 2 |

*Samples 10 and 16 not fully scanned.

**S2. Laminography**

Laminography is an available method in the Easytom S system (RX Solutions, France) a method designed for defect inspection in wide, flat samples. Scans were performed at 60 kV, 10 W, 166 µA with a 0.5 mm aluminum filter, a voxel size of 6.5 µm, and a small focal spot. The laminography data consists of radiographs obtained through lateral but not rotational shifts of the sample. Similar to the µCT data, the lamonigraphy scans of each tooth were preprocessed to create 3D datasets using the Xact software provided by manufacturer. Following reconstruction, the a pseudo 3D volume provides a partial in-plane contrast-enhanced representation of the stones, allowing for structural information to be extracted at increasing depths beneath the sample surface (Voropaev et al., 2016).

**Fig. S4.** Coronal pulp stone, (a) Longitudinal section of a crown showing the position of the coronal pulp stone. (b) Laminography and µCT images taken approximately 100 µm beneath the surface of the polished sample, where the red arrow highlights an area with lower mineral density, appearing darker. (c) Images at 300 µm depth, where the red arrow points to regions with higher mineral density, visible as bright white spots. (d) images at 500 µm depth, revealing dentinal tubules (red arrow). These internal structures are visible only with laminography and cannot be detected using µCT, note also in slide c.

**S3. Scanning electron microsopy and energy dispersive x-ray spectroscopy (EDX)**

To examine the microstructure, a Phenom XL was used to scan large pulp stone regions at high resolution. Using the SEM software, multiple images obtained across each sample were stitched together to create large fields of view. The backscatter image mode is sensitive to different densities and/or elemental compositional differences and maps were stitched together using ImageJ/Fiji.

To obtain spatially-resolved chemical information at µm resolution, 3 representative samples were scanned, uncoated, using a low-vacuum Phenom Pro X at 600x magnification (15 kV, 60 Pa, Thermofisher Scientific, Netherlands). The backscatter imaging mode helped to identify regions with lower and higher density. Point spectra with long counting times were collected in these regions as well as in a reference region in the adjacent healthy dentin. The chemical elements identified with Phenom Pro X software included oxygen, calcium, carbon, phosphorus, magnesium, sodium, potassium, and zinc.

EDX analysis made it possible to compare elemental composition, revealing that dense and lighter areas within the pulp stones shared elemental similarities with normal dentin. The EDX matched with SEM images highlighted the structural diversity of pulp stones, where some areas closely resembled dentin, supporting the idea that certain stones may have a dentin-like composition (Johnson and Bevelander, 1956). Analysis of the outer, brighter areas of pulp stones by EDX found no difference in the presence of trace elements, indicating increased mineral density without substitution of calcium by heavier elements. This observation aligns with findings reported in prior studies (Berès et al., 2016).

**Fig. S5.** EDX Analysis of (a) Coronal Pulp Stone and (b) Radicular Pulp Stone. The intensity profiles of various areas within the pulp stones are compared with the normal dentin structure. P: pulp stone, D: dentin.

**S4. Microfoculs X-ray Diffraction and Fluorescence**

The 200 µm thick parallel slices were placed in the center of a lateral translation frame accompanied by calibration stripes made of Al_2_O_3_. Thin sheets of polyimide tape were used to hold the samples and calibrants suspended in the frame. The X-ray microfocus beam had a diameter of 50 µm and an energy of 18 keV. Each sample was moved in both vertical and horizontal directions in the beam to map points within the samples in a grid with steps of 50 µm. The diffraction patterns were collected behind the sample with a Dectris Eiger X 9M area detector (Fig. S1), while XRF spectra were collected using a silicon drift XRF detector (RaySpec, Berlin) mounted at an angle to the sample.

**Fig. S6.** Schematic illustration of the pulp stone mapping experiment on the µSpot beamline at BESSY II (HZB, Berlin). Each pulp stone was mounted in a frame and laterally and horizontally scanned in the beam. Two kinds of detectors collect data with a beamsize of 50 µm diameter X-ray diffraction (XRD) and X-ray Fluorescence (XRF): inset (a) XRF spectrum. (b) pattern-ray diffractogram.

**S5. Low-vacuum lab µ-XRF**

We utilized a modified M4 Tornado µ-XRF spectrometer (Bruker Nano GmbH, Germany) equipped with a rhodium X-ray tube within the joint research group Synclab of the HZB at BLiX (Berlin Laboratory for innovative X-ray Technologies) at the Technical University of Berlin, Germany. The lateral resolution of the instrument is approximately 30 μm for Fe K-lines (Bauer et al., 2020). Measurements were conducted under vacuum (2–20 mbar) to enhance detection of light elements. The X-ray source was set to an acceleration voltage of 50 kV and a current of 1 mA. Overview maps were performed using a step size of 50 µm with an acquisition time of 1 second per pixel, whereas detailed measurements of pulp stones were performed using a step size of 10 µm and an acquisition time of 3 seconds per pixel. A 12.5 µm aluminium filter was applied in selected measurements. XRF spectra were deconvolved using the in-house software SpecFit (Förste et al., 2022) and converted into 2D elemental distribution maps, which were visualized using ImageJ (version 1.54f).

µ-XRF is a high-sensitivity, non-contact technique for analyzing the chemical composition of materials with high spatial resolution. Similar to the SEM-EDX method, emitted radiation is typically detected using an energy-dispersive silicon drift detector (SDD). The energy of the emitted fluorescence radiation is characteristic of specific elements, providing a unique 'fingerprint' that can be analyzed in a fluorescence spectrum. Using this method, which is more sensitive to PPM quantities of heavier elements compared to EDX, we mapped zinc in our samples. Zn is well known to play a role in mineralization and body defense mechanisms (Cho et al., 2007). Given its antibacterial properties and involvement in bone growth, Zn may have an important function in initiating or driving pulp stone formation.

Zinc has been identified as a key element bound to metalloenzymes in both bone (Gomez et al., 1999) and dentin (Deymier‐Black et al., 2014; Stock et al., 2014; Xue et al., 2008). Among these enzymes, matrix metalloproteinases (MMPs) are particularly noteworthy, as they rely on zinc for their function. MMPs play a critical role in the remodeling and breakdown of the extracellular matrix, which is essential for tissue repair and maintenance (Anumula et al., 2024). In the context of dental tissues, MMPs are involved in processes such as dentin formation and repair, where zinc contributes to the remineralization of dental hard tissues (Fatima et al., 2016; Ziyati et al., 2024). Zinc is known to play a protective role in maintaining tissue integrity (Lynch, 2011). This raises the hypothesis that zinc accumulation in pulp stones may not merely be a byproduct of mineralization but could instead represent an active biological response aimed at maintaining pulp health under stressful conditions. However, the exact mechanism by which it contributes to pulp stone formation remains unclear.

Figs. S5 and. S6 present additional detailed µ-XRF imaging of radicular and coronal pulp stones in a bovine tooth slice respectively. Elemental maps of Ca, P, Zn, Mg, and Na were acquired with 10 µm pixel size, 3 s dwell time, and 2 mbar vacuum. A Ca/P ratio map was generated by pixel-wise division of the deconvolved net-intensity maps, with low-signal pixels masked to avoid artefacts. The resulting elemental distributions highlighting the chemical heterogeneity of pulp stones and providing an expanded view of Zn variability compared to surrounding dentin. Due to the thickness of the specimen, µXRF signals partly originate from deeper regions of the pulp, producing a bright horizontal line artifact. The upper pulp stone was excluded from the corrected figure, as it was not in the same plane as the lower one, and retaining it could confuse interpretation.

**S6. widely accepted pulp stone insights based on historical studies**

The formation and characteristics of dental pulp stones have intrigued researchers since 1930s (Stafne and Szabo, 1933). The first 50 years of literature (1933–1988) (Johnson and Bevelander, 1956; Moss-Salentijn and Hendricks-Klyvert, 1988; Sayegh and Reed, 1968; Stafne and Szabo, 1933) established classification frameworks, debated mineralogical parallels with bone, and highlighted epidemiological patterns. These studies revealed that pulp stones are more prevalent in molars, particularly first molars, and that their incidence increases with age (Johnson and Bevelander, 1956; Moss-Salentijn and Hendricks-Klyvert, 1988; Sayegh and Reed, 1968). However, the advent of advanced imaging and spectroscopy has reshaped our understanding. CBCT studies (Kaabi et al., 2023) revealed their near-universal prevalence, while physicochemical analyses (Berès et al., 2018, 2016) linked trace elements like Zn and Cu to oxidative stress. Genetic insights (Berès et al., 2018) further distinguished pathological mineralization from inflammatory processes, and recent studies have positioned pulp stones as potential indicators of systemic conditions such as diabetes and hypertension (Khan et al., 2024).

Previous studies, such as those by Sayegh & Reed (1968) (Sayegh and Reed, 1968), underscored the profound influence of aging, with calcifications detected in nearly 90% of non-carious teeth from individuals over 45 years old—a stark contrast to the 8% prevalence in younger populations. Molars, particularly first molars, emerge as consistent epicenters for these calcifications, as evidenced by Kaabi et al. (2023) (Kaabi et al., 2023), who reported a 91% prevalence in first molars of Saudi population, imaged using CBCT imaging.

Premolars, however, exhibit markedly lower rates, with radiologic surveys such as Tarnes et al. (1982) (Tamse et al., 1982) reporting as little as 7.5% prevalence in mandibular premolars. These figures are likely underestimated due to the limitations of conventional radiography in detecting sub-200 μm calcifications. The prevalence of pulp stones varies across different populations. Sener et al. (Şener et al., 2009) found that 0.4% of patients had pulp stones in 12 or more teeth, while Deniz et al. (Deniz and Oz, 2023) reported this occurrence in 1.6% of patients. Both studies focused on permanent teeth, with Sener et al. [39] examining adult patients and Deniz et al. (Deniz and Oz, 2023) analyzing a pediatric cohort.

Pulp stones increasingly serve as biomarkers of systemic conditions. Khan et al. (2024) (Khan et al., 2024) linked their presence to diabetes and hypertension, with 88.5% of diabetic patients exhibiting these calcifications. Meanwhile, advancements in imaging, exemplified by Lv et al.’s (2024) (Lv et al., 2024) CBCT-driven case of idiopathic pulp stones in mixed dentition, challenge long-held assumptions about caries or trauma as sole etiologic drivers.

Pulp stone formation can occur in deciduous or permanent, healthy or diseased, and in unerupted or impacted teeth (Moss-Salentijn and Hendricks-Klyvert, 1988; Sayegh and Reed, 1968). Though possibly associated with triggering trigeminal neuralgia (Zhang et al., 2024), generally speaking, pulp mineralization does not affect the pulp vitality (Bonilla-Represa et al., 2021).

**S7. Clinical implications and Future Directions**

Our findings have significant clinical implications, primarily concerning the detection and management of pulp stones during endodontic procedures. The high prevalence of these mineralizations, even in young, caries-free teeth, suggests they are a common yet often overlooked clinical feature. We demonstrate a critical diagnostic gap: while larger, attached stones may be identified on clinical X-rays or CBCT, smaller 'free-standing' stones are frequently missed by these methods. The superior resolution of µCT revealed that these smaller stones can be numerous, raising the possibility that their true prevalence in human teeth is underestimated by routine imaging.

Undetected pulp stones can lead to unforeseen dental treatment complications. Large mineral masses can obstruct the pulp chamber, complicating canal location and negotiation. Smaller, free-floating stones pose a different risk: they may be overlooked on preoperative scans and become lodged near the apex during instrumentation, potentially contributing to treatment failures by harboring contaminants. Whether such residual calcifications are a direct cause of post-treatment disease warrants further investigation. To aid in managing these challenges, our observation of a distinct color contrast between pulp stones and surrounding dentin may be clinically valuable. This color difference, often visible with the endodontic microscopes used in complex cases, could serve as a visual guide for clinicians attempting to remove obstructive mineralizations (Krasner and Rankow, 2004). Beyond the endodontic challenges they present, understanding the biological mechanisms of pulp stone formation could inform future regenerative strategies. The processes driving this rapid, localized mineralization might be harnessed for applications like pulp capping, where stimulating new, protective hard tissue is the goal. Translating these findings from a bovine model to clinical practice will, however, require further long-term studies.

**Supplementary Video 1**: Three-dimensional visualization of a bovine incisor using cone-beam computed tomography (CBCT), highlighting the limited spatial resolution and reduced ability to detect fine details within the pulp chamber.

**Supplementary Video 2**: High-resolution micro-computed tomography (μCT) visualization of the same bovine incisor shown in Video 1. The scan reveals significantly more anatomical detail, clearly delineating pulp stones and internal features within the pulp chamber.

**Supplementary Video 3:** Laminographic visualization of a coronal pulp stone. Dynamic cross-sectional imaging reveals concentric internal layering and subtle mineral density variations not detectable by standard μCT, indicating structural heterogeneity and potential remnants of organic matrix.

**Supplementary Video 4:** Laminographic visualization of a radicular pulp stone. The video reveals pronounced internal channel-like structures and alternating high- and low-density domains, supporting the interpretation of non-uniform mineralization and possible organic inclusions.

Reference

Anumula, L., Ramesh, S., Kolaparthi, V.S.K., 2024. Matrix metalloproteinases in dentin: Assessing their presence, activity, and inhibitors–a review of current trends. Dent. Mater.

Bauer, L.J., Mustafa, H.A., Zaslansky, P., Mantouvalou, I., 2020. Chemical mapping of teeth in 2D and 3D: X-ray fluorescence reveals hidden details in dentine surrounding fillings. Acta Biomater. 109, 142–152.

Berès, F., Isaac, J., Mouton, L., Rouzière, S., Berdal, A., Simon, S., Dessombz, A., 2016. Comparative physicochemical analysis of pulp stone and dentin. J. Endod. 42, 432–438.

Berès, F., Lignon, G., Rouzière, S., Mauprivez, C., Simon, S., Berdal, A., Dessombz, A., 2018. Physicochemical analysis of human pulpal mineralization secondary to FAM20A mutations. Connect. Tissue Res. 59, 46–51.

Bonilla-Represa, V., Gil-Flores, J., López-Frías, F.J., Abalos-Labruzzi, C., Guerrero-Belizón, E., Herrera-Martínez, M., 2021. Analysis on the predictive value of different variables in pulp stones appearance frequency and its pulpal response to cold stimuli. Odontology 109, 321–326.

Cho, Y.-E., Lomeda, R.-A.R., Ryu, S.-H., Sohn, H.-Y., Shin, H.-I., Beattie, J.H., Kwun, I.-S., 2007. Zinc deficiency negatively affects alkaline phosphatase and the concentration of Ca, Mg and P in rats. Nutr. Res. Pract. 1, 113–119.

Deniz, I., Oz, E., 2023. The prevalence of pulp stones in a Turkish paediatric cohort from the Isparta district: an 8-year retrospective radiographic study. Eur. Arch. Paediatr. Dent. 24, 729–736.

Deymier‐Black, A.C., Veis, A., Cai, Z., Stock, S.R., 2014. Crystallographic texture and elemental composition mapped in bovine root dentin at the 200 nm level. Scanning J. Scanning Microsc. 36, 231–240.

Fatima, T., Rahim, Z., Lin, C.W., Qamar, Z., 2016. Zinc: A precious trace element for oral health care. J Pak Med Assoc 66, 1019–1023.

Förste, F., Bauer, L., Heimler, K., Hansel, B., Vogt, C., Kanngießer, B., Mantouvalou, I., 2022. Quantification routines for full 3D elemental distributions of homogeneous and layered samples obtained with laboratory confocal micro XRF spectrometers. J. Anal. At. Spectrom. 37, 1687–1695.

Gomez, S., Rizzo, R., Pozzi-Mucelli, M., Bonucci, E., Vittur, F., 1999. Zinc mapping in bone tissues by histochemistry and synchrotron radiation–induced X-ray emission: correlation with the distribution of alkaline phosphatase. Bone 25, 33–38.

Johnson, P.L., Bevelander, G., 1956. Histogenesis and histochemistry of pulpal calcification. J. Dent. Res. 35, 714–722.

Kaabi, H.H., Riyahi, A.M., Al-Maflehi, N.S., Alrumayyan, S.F., Bakrman, A.K., Almutaw, Y.A., 2023. Three-dimensional analysis for detection of pulp stones in a Saudi population using cone beam computed tomography. J. Oral Sci. 65, 257–260.

Khan, M.I., Khare, A., Arif, K., Khan, S.S., Nasir, A., Lari, S., 2024. Dental pulp stones and their correlation with metabolic diseases. J. Oral Maxillofac. Pathol. 28, 192–199.

Lv, Y., Zhu, J., Fu, C.-T., Liu, L., Wang, J., Li, Y.-F., 2024. Multiple pulp stones emerge across all teeth during mixed dentition: A case report. World J. Clin. Cases 12, 5189.

Lynch, R.J.M., 2011. Zinc in the mouth, its interactions with dental enamel and possible effects on caries; a review of the literature. Int. Dent. J. 61, 46–54.

Moss-Salentijn, L., Hendricks-Klyvert, M., 1988. Calcified structures in human dental pulps. J. Endod. 14, 184–189.

Sayegh, F.S., Reed, A.J., 1968. Calcification in the dental pulp. Oral Surgery, Oral Med. Oral Pathol. 25, 873–882.

Şener, S., Cobankara, F.K., Akgünlü, F., 2009. Calcifications of the pulp chamber: prevalence and implicated factors. Clin. Oral Investig. 13, 209–215.

Stafne, E.C., Szabo, S.E., 1933. The significance of pulp nodules. Dent Cosm. 75, e4.

Stock, S.R., Deymier-Black, A.C., Veis, A., Telser, A., Lux, E., Cai, Z., 2014. Bovine and equine peritubular and intertubular dentin. Acta Biomater. 10, 3969–3977.

Tamse, A., Kaffe, I., Littner, M.M., Shani, R., 1982. Statistical evaluation of radiologic survey of pulp stones. J. Endod. 8, 455–458.

Voropaev, A., Myagotin, A., Helfen, L., Baumbach, T., 2016. Direct Fourier inversion reconstruction algorithm for computed laminography. IEEE Trans. Image Process. 25, 2368–2378.

Xue, J., Zhang, L., Zou, L., Liao, Y., Li, J., Xiao, L., Li, W., 2008. High-resolution X-ray microdiffraction analysis of natural teeth. J. Synchrotron Radiat. 15, 235–238.

Zhang, W., Wang, Y., Ye, L., Zhou, Y., 2024. Distribution and influencing factors of pulp stones based on CBCT: a retrospective observational study from southwest China. BMC Oral Health 24, 947.

Ziyati, O., Abou-Obeida, S., Mtalai, N., El Boussiri, K., 2024. Zinc and Oral Health: Literature Review. Open Access Libr. J. 11, 1–23.
